# Dataset on application of electrochemical and photochemical processes for sulfacetamide antibiotic elimination in water

**DOI:** 10.1016/j.dib.2020.105158

**Published:** 2020-01-21

**Authors:** Gina Hincapié-Mejía, Fidel Granda-Ramírez, Franklin Ferraro, Efraím A. Serna-Galvis, Ricardo A. Torres-Palma

**Affiliations:** a Grupo de Investigación Ambiente, Hábitat y Sostenibilidad, Facultad de Arquitectura e Ingeniería, Institución Universitaria Colegio Mayor de Antioquia (IUCMA), Carrera 78 No. 65-46, Medellín, Colombia; b Departamento de Ciencias Básicas, Universidad Católica Luis Amigó, Transversal 51A No. 67B-90, Medellín, Colombia; c Grupo de Investigación en Remediación Ambiental y Biocatálisis (GIRAB), Instituto de Química, Facultad de Ciencias Exactas y Naturales, Universidad de Antioquia UdeA, Calle 70 No. 52-21, Medellín, Colombia

**Keywords:** Antibiotics degradation, Degradation routes, DFT analysis, Matrix effects, Treatment extent

## Abstract

Sulfonamide-class antibiotics are recognized as water pollutants, which have negative environmental impacts. A strategy to deal with sulfonamides is throughout the application of oxidation processes. This work presents the treatment of the sulfacetamide (SAM) antibiotic by electrochemical oxidation, UV-C/H_2_O_2_ and photo-Fenton process. It was established the main degradation routes during each process action. A DFT computational analysis for SAM structure was done and mass spectra of primary transformation products were determined. Chemical oxygen demand (COD), total organic carbon (TOC) and biochemical oxygen demand (BOD_5_) were also followed. Additionally, SAM treatment in simulated seawater and hospital wastewater was measured. These data can be useful for comparative purposes about degradation of sulfonamide-class antibiotics by electrochemical and advanced oxidation processes.

**Table:** Specifications Table

Subject	Environmental chemistry, Chemical Engineering
Specific subject area	Electrochemical and advanced oxidation processes
Type of data	Table Figure Picture
How data were acquired	Data were acquired by using HPLC, HPLC-MS and Gaussian software
Data format	Raw Analyzed
Parameters for data collection	The experimental tests were developed at fixed conditions to evaluate the ability of processes to eliminate a representative sulfonamide antibiotic
Description of data collection	All experimental data were obtained at lab-scale
Data source location	Institución Universitaria Colegio Mayor de Antioquia (IUCMA), Universidad de Antioquia and Universidad Católica Luis Amigó, Medellín, Colombia
Data accessibility	Mendeley data repository through the following link: https://data.mendeley.com/datasets/bctc4dh2ck/draft?a=cd8f77df-08f8-45d9-ae48-1f9f8186ac9c

**Table:** 

**Value of the Data** • Data show similarities and differences among the processes regarding degradation routes, primary transformation products, indicators such as COD, TOC and BOD_5_; and matrix effects during SAM treatment. • Data can benefit people working on treatment of wastewaters containing antibiotics. • Data can be useful for comparative purposes about elimination of antibiotics by electrochemical and advanced oxidation processes. • Data could be useful for scaling up of the process to treat organic pollutants in water. • Data may be utilized in further theoretical and experimental researches on oxidation of sulfonamides by electrophilic species.

## 1 Data description

**Fig. 1:**
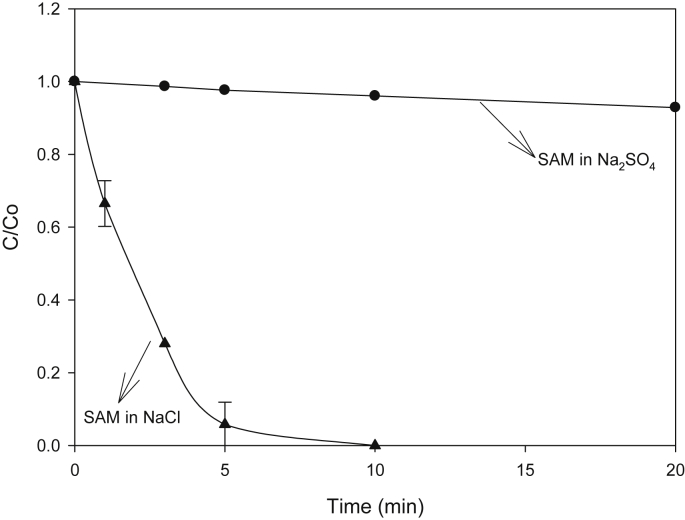
Electrochemical treatment of SAM.

Information on the main degradation routes for sulfacetamide (SAM) treatment by the considered processes is initially presented. Such data are relevant to understand action of the systems on the antibiotics [1]. The considered electrochemical system is characterized by the action of active chlorine species as degrading agents (mediated route, Eqs (1), (2), (3), (4)), in NaCl presence. Meanwhile, when Na_2_SO_4_ is used as supporting electrolyte, oxidative species in solution bulk cannot be generated from sulfate ions, but oxidation on anode surface (direct route) can be evidenced [[2], [3], [4]]. Fig. 1A depicts degradation of the sulfonamide by electrochemical oxidation utilizing two supporting electrolytes (i.e., NaCl and Na_2_SO_4_) to identify the action routes of the system.

(1) Ti/IrO_2(anode)_ + 2Cl^−^ → Cl_2_ + 2e^−^

(2) Cl_2_ + H_2_O → HOCl + HCl

(3) HOCl + H_2_O → H_3_O^+^ + OCl^−^

(4) Cl_2_, HOCl, OCl^−^ + organic pollutant → degradation products

**Fig. 2:**
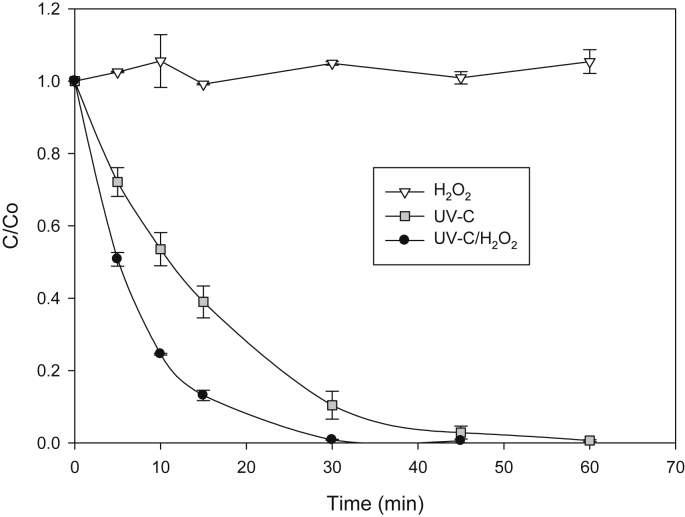
Degradation of SAM by UV-C/H_2_O_2_.

Fig. 2 presents evolution of SAM under UV-C irradiation, hydrogen peroxide and AOP UV-C/H_2_O_2_; this last system generates hydroxyl radical (Eq. (5)) as main degrading species (Eq. (6)) [5].

(5) UV-C + H_2_O_2_ → 2 HO•

(6) HO• + organic pollutant → degradation products

**Fig. 3:**
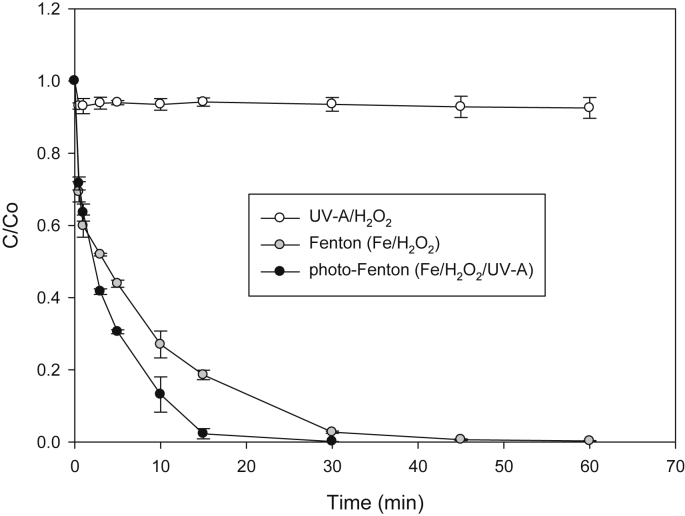
Evolution of SAM during treatment by photo-Fenton.

In Fig. 3 is shown SAM elimination by photo-Fenton system (which involves interaction of iron ions with hydrogen peroxide and light to produce HO•, Eqs. (7), (8) [6]). Control experiments (i.e., UV-A/H_2_O_2_ and Fenton) are also presented in Fig. 3.

(7) Fe^2+^ + H_2_O_2_ → Fe^3+^ + HO• + HO^−^

(8) Fe^3+^ + H_2_O + *hν*_*(UV–vis)*_ → Fe^2+^ + HO• + H^+^

**Fig. 4:**
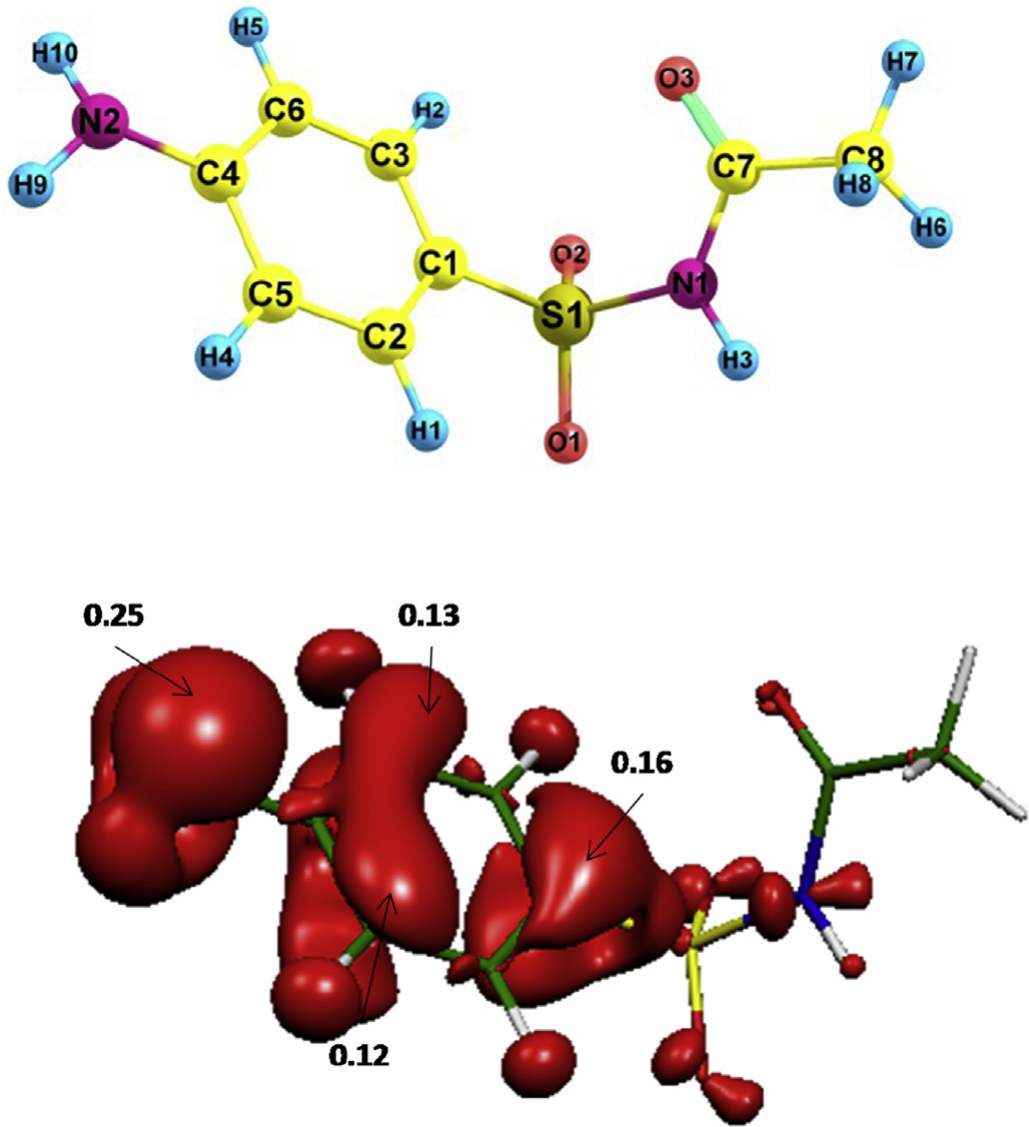
Electron density regions on SAM more susceptible to attacks by electrophilic species (e.g., HOCl or HO•).

**Fig. 5:**
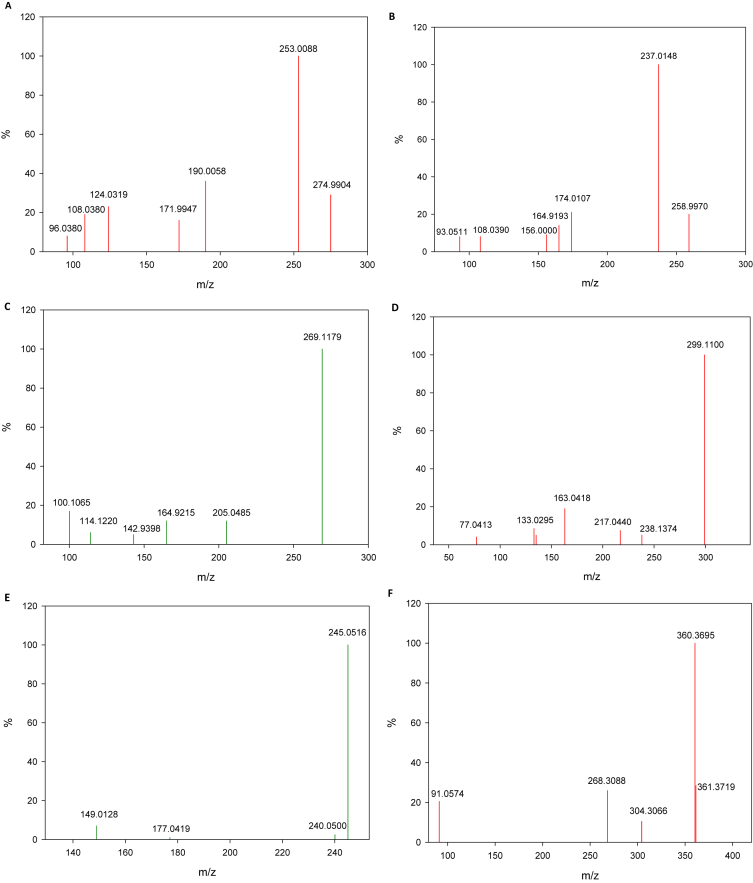
Mass spectra of primary degradation products.

Degradation of SAM under the oxidation processes can be promoted by active chlorine (e.g., electrochemistry) or hydroxyl radical (e.g., photo-Fenton and UV-C/H_2_O_2_). These are electrophilic species able to attack electron rich moieties [1,4]. Then, computational calculations to identify regions on SAM with high electron density and susceptible to attacks by such degrading agents was carried (Fig. 4). Additionally, to determine the primary transformations, analyses of HPLC-MS were performed. Table 1 summarizes the primary products found for SAM treatment by each process; whereas, Fig. 5 contains mass spectra of the transformation products.

**Table 1:** Summary of primary products found for SAM treatment by the considered processes.

Product [M+H]^+^ (*M*ass *spectrum in*Fig. 5*)*	Process			
	Electrochemical oxidation	UV-C alone	UV-C/H_2_O_2_	Photo-Fenton
275 (*A*)			X	
259 (*B*)	X	X	X	X
269 (*C*)		X		
299 (*D*)	X		X	
245 (*E*)	X	X	X	X
360 (*F*)	X			

X means the product was found during degradation by the process.

**Fig. 6:**
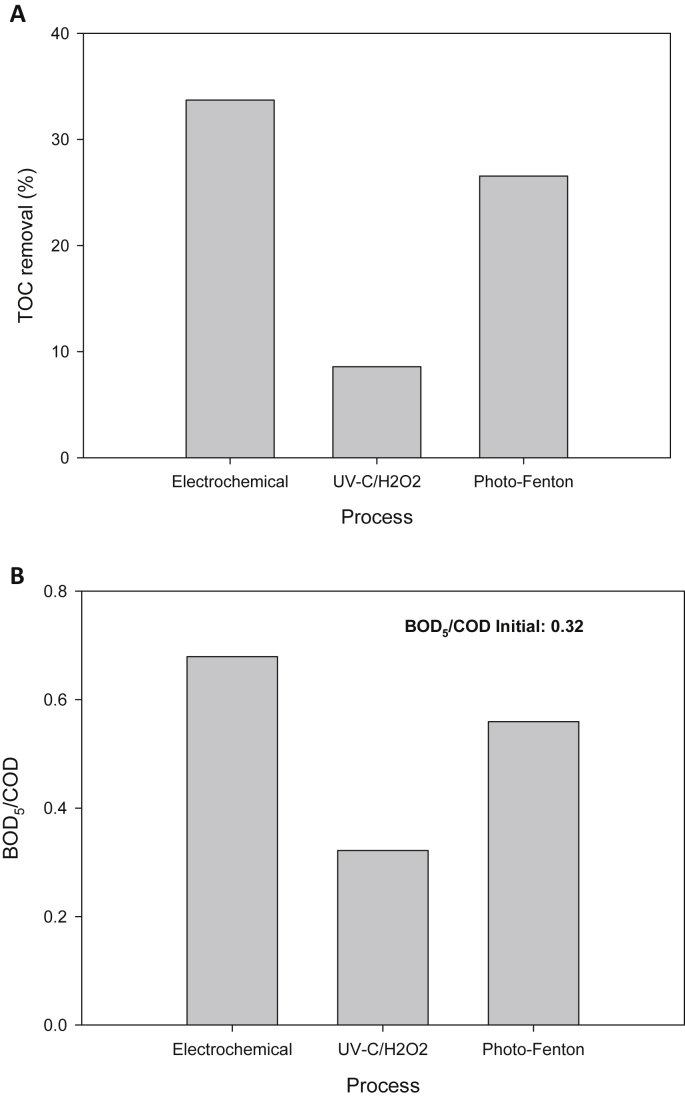
Treatments extent.

To establish the treatments extent, COD, TOC and BOD_5_ were measured at 100% of SAM degradation by each considered process. Fig. 6A illustrates the TOC removal, whereas Fig. 6B presents the biodegradability relationship (BOD_5_/COD).

**Fig. 7:**
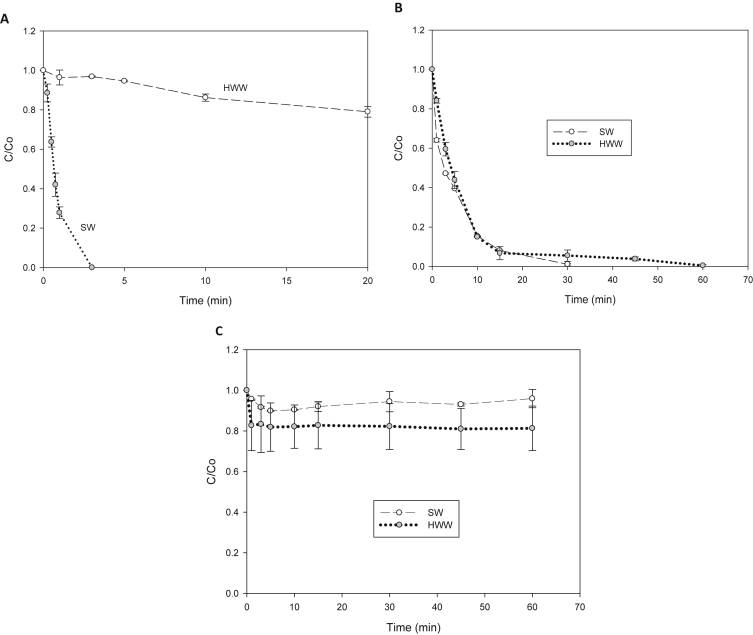
Degradation of SAM in simulated seawater (SW) and hospital wastewater (HWW) by the diverse processes. **A.** Electrochemistry. **B.** UV-C/H_2_O_2_. **C.** Photo-Fenton.

To test the ability of the processes to degrade the antibiotic in complex matrices, treatment of SAM in simulated seawater and hospital wastewater (see composition in Table 2) was performed. Fig. 7 shows the removal of SAM in these complex matrices during treatment by the three oxidation systems.

**Table 2:** Composition of the complex matrices.

Seawater (SW)^a^								
Component	NaCl	MgSO_4*_7 H_2_O		CaCl_2*_2H_2_O		NaHCO_3_		pH
Concentration (μmol L^−1^)	500000	50000		10000		2000		6.5
Hospital wastewater (HWW)^a^								
Component	NaCl	CaCl_2_	KCl	Na_2_SO_4_	KH_2_PO_4_	NH_4_Cl	Urea	pH
Concentration (μmol L^−1^)	51300	340	1340	710	370	940	21000	6.5

a Matrix prepared according to Refs. [7,8].

The raw data for the above figures and tables is available on the Mendeley data repository (see this link: https://data.mendeley.com/datasets/bctc4dh2ck/draft?a=cd8f77df-08f8-45d9-ae48-1f9f8186ac9c).

## 2 Experimental design, materials, and methods

### 2.1 Reagents

Sodium sulfacetamide was provided by Corpaul (Medellín, Colombia) Sodium chloride, calcium chloride dihydrate, potassium chloride, ammonium chloride, sodium sulfate, potassium dihydrogen phosphate, urea and acetonitrile were purchased from Merck (Darmstadt, Germany). Urea was purchased from Carlo Erba (Sabadell, Spain) and formic acid was provided by Carlo-Erba (Val de Reuil, France). All chemicals were used as received. The solutions were prepared using distilled water.

### 2.2 Reaction systems

An electrolytic cell equipped with a Ti/IrO_2_ rectangular plate of 8 cm^2^ (anode, previously characterized [9]), a zirconium spiral of 10 cm^2^ (cathode) and a WK electric apparatus (as current source) were used for the electrochemical experiments (Picture 1). A current density of 5 mA cm^−2^ and 0.05 mol L^−1^ of NaCl or Na_2_SO_4_ as supporting electrolyte were used. The electrochemical system was operated under constant stirring conditions. In the experiments, 150 mL of the antibiotic solutions were treated. Each experiment was performed at least by duplicate. During treatments, aliquots of 1.2 mL were taken at regular time intervals to perform the analyses of antibiotic evolution.

**Picture 1:**
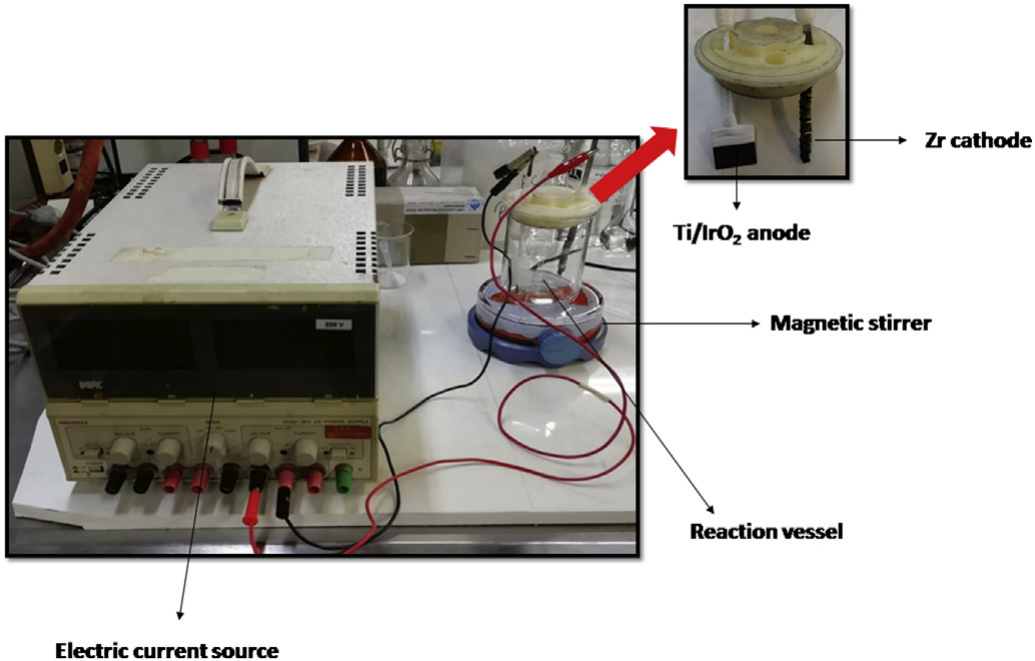
Electrochemical reaction system.

The photochemical processes were carried out in a homemade aluminum reflective reactor. In the case of UV-C/H_2_O_2_, the reaction system was equipped with 5 UV-C lamps (LUMEK T8 15W) with main emission at 254 nm (Picture 2). Antibiotic (40 μmol L^−1^) solutions (150mL) were placed in beakers under constant stirring. Each experiment was performed at least by duplicate. During treatments, until eight aliquots of 1.0 mL were taken at regular time intervals to perform the analyses.

**Picture 2:**
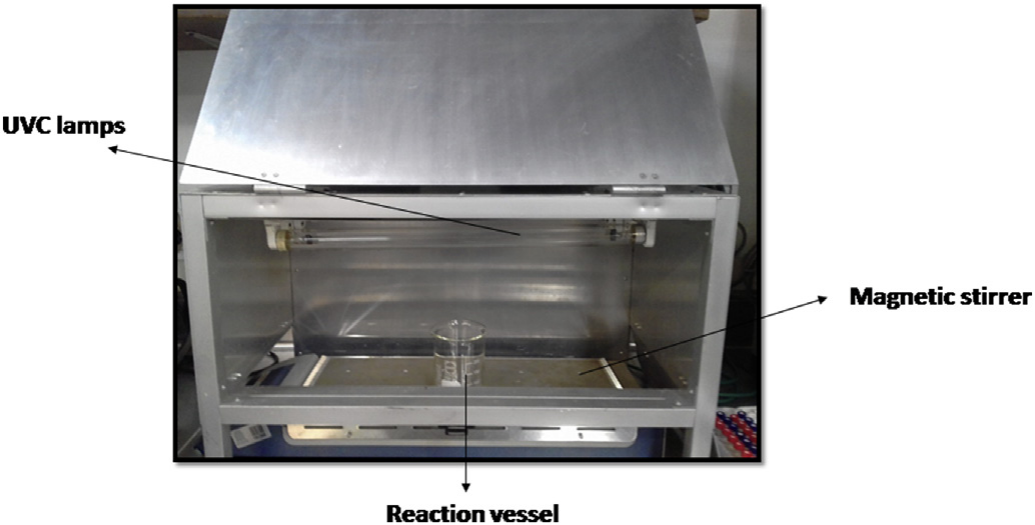
Reaction system for UV/PS process.

Meanwhile, for photo-Fenton process, the same homemade aluminum reflective reactor equipped with 5 lamps (LuxTech T8 15W) was utilized. SAM (40 μmol L^−1^) solution (150 mL) was placed in beakers under constant stirring. In the photo-Fenton process, 500 μmol L^−1^ and 45 μmol L^−1^ of H_2_O_2_ and Fe (II) respectively were used.

### 2.3 Analyses

The evolution of SAM during treatments was followed by sing a UHPLC Thermoscientific Dionex UltiMate 3000 instrument equipped with an Acclaim™ 120 RP C18 column (5 μm, 4.6 × 150 mm) and a diode array detector. The injection volume was 20 μL, and a mixture of acetonitrile/aqueous formic acid (10 mmol L^−1^, pH 3.0) 40/60% V/V was used as mobile phase. The UV detection was carried out at 257, 270, 280 and 290 nm. It must be indicated that all degradation experiments were carried out at least by duplicate.

The computational analysis was performed with the Fukui function by applying the framework of functional density theory (DFT). SAM structure was optimized with the B3LYP hybrid functional density [10], with the 6-31 + G* basis set and the continuous polarization model [11] using the dielectric constant for water. Thus, **f**^**-**^ (i.e., electrophilic Fukui functions) values were calculated. For **f**^**-**^, a higher number is an indicator of a higher possibility of attack by electrophilic species.

The determination of primary transformation products was carried out at 50% of the antibiotic degradation. For such determination an ACQUITY UPLC H-Class (Waters Corporation, Milford, MA, USA) equipped with a quaternary solvent supply manager and a sampler manager coupled to an Xevo-G2-XS-Q-Tof, Mass spectrometer equipped with an electrospray interface (Waters Corporation). A Restek C18 column (50 × 2.1 mm; 1.7 μm) was used with water (acidified by 0.1% formic acid) and acetonitrile (ACN) as eluents. The flowrate was 0.5 mL min^−1^ at room temperature. The gradient was from 90/10% water/ACN until 2 min, then the gradient change to 75/25% to 3 min, in 3,5 min change again to 90/10% to 6 min.

ESI + positive ionization mode and a sensitivity analysis were used for MS Determination with an analysis range of 50–700 Da, with a scan time of 0.1 s and a delay between operations of 0.01 s, for an analysis time of 6 minutes. The collision energy ramp was 10–30 V and the cone voltage was 30 V.

Chemical oxygen demand (COD) was established according to the Standard Methods for Examination of Water and Wastewater (5220 D). The closed reflux colorimetric method was used. An aliquot of 2500 μL of sample was added to a digestion vessel containing 1500 μL of digestion solution (potassium dichromate in concentrated sulfuric acid) and 3500 μL of sulfuric acid reagent (silver sulfate in concentrated sulfuric acid). The digestion was performed at 140 °C during 2 hours in a Velp Thermo-reactor. Absorbance was measured at 600 nm in a Lab Scient UV-1100 spectrophotometer.

Biochemical oxygen demand at 5 days (BOD_5_) was carried out according to the Standard Methods for Examination of Water and Wastewater (5210 B) using an Oxitop respirometric system thermostatted at 20 °C. The volume added to the incubation bottle contained 270 mL (10% V/V was the inoculum and 90% V/V was the sample). Prior to analysis, the pH was adjusted to near neutrality using sodium hydroxide (1.0 M), and residual hydrogen peroxide or active chlorine species were eliminated using sodium bisulfite (0.1 M).

Total organic carbon (TOC) was measured using a Teledyne Tekmar TOC analyzer. This was determined by combustion with catalytic oxidation at 680 °C using high-purity oxygen gas at a flow rate of 190 mL min^−1^. The apparatus had a non-dispersive infrared detector. Calibration of the analyzer was attained with standard potassium hydrogen phthalate (99.5%) solution. The injection sample volume was 50 μL.
